# Functionally Equivalent Variants in a Non-standard Variety and Their Implications for Universal Grammar: A Spontaneous Speech Corpus

**DOI:** 10.3389/fpsyg.2017.01260

**Published:** 2017-07-25

**Authors:** Evelina Leivada, Elena Papadopoulou, Natalia Pavlou

**Affiliations:** ^1^Department of English Studies, University of Cyprus Nicosia, Cyprus; ^2^Cyprus Acquisition Team Nicosia, Cyprus; ^3^School of Humanities and Social Sciences, European University Cyprus Engomi, Cyprus; ^4^Department of Linguistics, University of Chicago, Chicago IL, United States

**Keywords:** variation, dialect, bilingualism, Universal Grammar, parameters, falsifiability

## Abstract

Findings from the field of experimental linguistics have shown that a native speaker may judge a variant that is part of her grammar as unacceptable, but still use it productively in spontaneous speech. The process of eliciting acceptability judgments from speakers of non-standard languages is sometimes clouded by factors akin to prescriptive notions of grammatical correctness. It has been argued that standardization enhances the ability to make clear-cut judgments, while non-standardization may result to grammatical hybridity, often manifested in the form of functionally equivalent variants in the repertoire of a single speaker. Recognizing the importance of working with corpora of spontaneous speech, this work investigates patterns of variation in the spontaneous production of five neurotypical, adult speakers of a non-standard variety in terms of three variants, each targeting one level of linguistic analysis: syntax, morphology, and phonology. The results reveal the existence of functionally equivalent variants across speakers and levels of analysis. We first discuss these findings in relation to the notions of competing, mixed, and fused grammars, and then we flesh out the implications that different values of the same variant carry for parametric approaches to Universal Grammar. We observe that intraspeaker realizations of different values of the same variant within the same syntactic environment are incompatible with the ‘triggering-a-single-value’ approach of parametric models, but we argue that they are compatible with the concept of Universal Grammar itself. Since the analysis of these variants is ultimately a way of investigating the status of Universal Grammar primitives, we conclude that claims about the alleged unfalsifiability of (the contents of) Universal Grammar are unfounded.

## Introduction

Research in non-standard varieties has reliably shown that the process of eliciting acceptability judgments from native speakers of such varieties —often called (non-standard) dialects— faces various challenges. Among them, one finds (i) the interference from prescriptive notions of correctness, that is, the outcome of speakers’ awareness that some of the variants of their native linguistic repertoire are considered ‘incorrect’ by speakers of the standard variety, (ii) a greater degree of interspeaker and intraspeaker variation due to non-standardization leading to less clear-cut variants and judgments over variants, and (iii) the unclear dividing lines among the various ‘lects’ (e.g., acrolect, mesolects, basilect) that exist on the standard-dialect continuum (Cheshire and Stein, 1997; Milroy, 2001; Henry, 2005; Papadopoulou et al., 2014). Such features blur the boundaries of grammatical variants in a way that results in a high degree of grammatical hybridity attested in the form of utterances that may incorporate elements from various lects *without* code-switching being in place (Cornips, 2006; Tsiplakou et al., 2016; Leivada and Grohmann, 2017).^1^ In this context, it has been argued that working from corpora of spontaneous speech might be more useful or desirable than using acceptability judgements when the language under investigation is a non-standard/-codified one — as is the case of the variety investigated in this study — because speakers may be influenced by prescriptive notions of correctness (Henry, 2005).

Findings from the field of experimental linguistics stress the necessity for corpora studies. The use of spontaneous speech corpora allows us to obtain reliable insights into speakers’ actual production instead of what they think or say they produce, which is possibly subject to more interference by prescriptive rules of language. It has been shown that native speakers may judge a grammatical variant as unacceptable, but still be recorded producing it spontaneously (Labov, 1996; Cornips and Poletto, 2005; Beltrama, 2013).^2^ If this is true in cases of monolingual speakers, in cases of bilingual or bilectal development (i.e., two varieties of the same language instead of two different languages) that at times involves non-standard/-codified —and as such, possibly more hybrid— varieties, *more* discrepancy is expected between speakers’ introspective judgments about their repertoire and the actual linguistic repertoire itself (Leivada et al., 2017). To explain this further, let’s consider Labov’s (1975, 1996) *Consensus Principle* in (I):

(I) The Consensus Principle
  If there is no reason to think otherwise, assume that the judgments of any native speaker are characteristic of all speakers of the language. (Labov, 1996: 79)

The Consensus Principle presupposes some degree of uniformity in terms of judgments among native speakers of the same language. However, when eliciting introspective judgments, other extra-grammatical factors and variables may interact with the linguistic performance in the phenomena under investigation, especially so when these judgments come from dialect speakers. Considering that (i) standardization leads to more clear-cut judgments and (ii) the possible emergence of various mesolects in the dialect-standard continuum which may feature different exponents/values for the same linguistic variant, it is possible that speakers of non-standard varieties will *not* be as uniform in terms of their judgments as the idealized picture of linguistic uniformity among the members of a linguistic community suggests (see also Chomsky, 1965). The existence of a dialect-standard continuum where varieties do not always appear with discrete edges invests the process of linguistic development and its outcome with an additional layer of complexity (Papadopoulou et al., 2014). Moreover, although acceptability judgment tasks are a reliable tool in linguistic research (Sprouse and Almeida, 2012; Sprouse et al., 2013), it has been noted that at times discrepancies can be observed between overt linguistic behavior and introspections about decontextualized, constructed examples, and these discrepancies are particularly pronounced when dialectologists or sociolinguists present data from non-standard dialects (Bresnan, 2007, see also Baggio et al., 2012 for a review). The following passage in Devitt (2006) illustrates how formal instruction and standardization may mediate the process of providing intuitive judgments about grammatical variants:

“As a graduate student, I spent a summer in the Pyrenees (Andorra, Perpign[a]n, etc.) doing field research on the phonology of various dialects of Catalan. Many of our native informants were illiterate peasants. I was forcefully struck how difficult it was to elicit linguistic judgments from them regarding their language, which of course they spoke perfectly well. Just getting the plurals of certain nouns was tough. These folks seemed to be very hard of hearing when it came to hearing the voice of competence! Their difficulty, it seemed, was that their native language was largely transparent to them—they had never thought of it as an object for observation and hence were largely unable to form even the most rudimentary judgments about its character. Catalan speakers with only a modicum of grade school education, by contrast, were good informants, presumably because they had learned through their grammar lessons to think of *language as an object with various properties*, even if they had no sophisticated knowledge of what those properties might be, theoretically speaking. (Bob Matthews, in correspondence).” (Devitt, 2006: 497)

Hagoort (2014) has recently made some useful suggestions with respect to the way linguistic work could increase its impact and visibility within cognitive (neuro)science. The first suggestion he offers is to exploit the availability of large corpora, the existence of which “puts linguists in a historically unprecedented position” (Hagoort, 2014). For these and other reasons, spontaneous speech data have been described as the best data that one can obtain for the study of language variation (Cornips, 2015). Recognizing the value of working with corpora in experimental linguistics, this work investigates patterns of grammatical variation and hybridity through analyzing the spontaneous production of five neurotypical, adult speakers of a non-standard variety in terms of three variants, each targeting a different level of linguistic analysis: syntax, morphology, and phonology. The variety under investigation is Cypriot Greek, a largely understudied language in many domains of grammar that lacks the status of an official language. Instead, Standard Modern Greek is the official, standard variety used in Cyprus, which shows the characteristics expected to find in such context, such as its use in school education, formal speech, and media.

**FIGURE 1:**
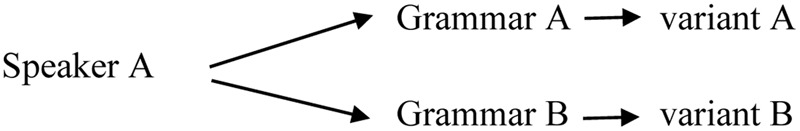
Functionally equivalent variants (Rosenbach, 2002, p. 82).

Apart from gaining valuable insights into the grammatical system of an understudied variety, investigating the limits of variation *within* a hybrid ‘lect’ is directly related to a parametric conception of Universal Grammar (UG) within the Principles & Parameters framework (Chomsky, 1981). The reason is the notion of *functionally equivalent variants* (Kroch, 1994) which, in the case of Cypriot Greek, is the result of (once) competing grammars.^3^ These variants are doublets that consist of two equivalent forms or constructions that have the exact same function, but are grammatically incompatible (**Figure 1**). Incompatibility here refers to the fact that the two variants A and B cannot co-exist in a single environment. As correctly pointed out in Embick (2008), the occurrence of functionally equivalent variants in the repertoire of a speaker poses important questions for the models through which language is interpreted.

After presenting the methodological aspects of this research and the obtained results in the next two sections, we discuss the implications that our findings carry for the notions of competing, mixed, and fused grammars as well as for parametric approaches to UG.

## Language Under Investigation

Cypriot Greek has often been referred to as a dialect of Greek (Contossopoulos, 2000); a variety that is linguistically proximal to Standard Modern Greek (Grohmann and Kambanaros, 2016; Grohmann et al., 2016), which is the official language in the environment our participants acquire language. Although the official language in education and other formal settings is indeed Standard Modern Greek, research has shown the boundaries between the two varieties, Standard Modern Greek and Cypriot Greek, and their distribution across different registers is not straightforward (Grohmann and Leivada, 2012; Tsiplakou et al., 2016). At times mixing is attested without code-switching being in place, while no official characterization has been provided for any of these terms in this specific context. The question arising in this context is whether the attested variants emerging in mixed speech repertoires are functionally equivalent for an individual speaker.

The two varieties have differences in all levels of linguistic analysis and often monolingual speakers of Standard Modern Greek judge Cypriot Greek as unintelligible. At the same time, Greek Cypriot speakers do not always provide reliable judgments of their own speech since these are often clouded by sociolinguistic attitudes toward using the non-standard variety. Cypriot Greek lacks official codification and its status as a different language/variety is often denied by Greek Cypriots who may downplay the differences between Standard Modern Greek and Cypriot Greek and describe the latter as just an accent (Arvaniti, 2010). As the discussion of the different variants will make clear in the next section, the two varieties have differences across levels of linguistic analysis and these differences vastly exceed the sphere of phonetics or phonology.

All speakers of Cypriot Greek have exposure to Standard Modern Greek through education and other mediums and in this way, they are competent to different degrees in both varieties. We employ the term ‘bilectal’ (Rowe and Grohmann, 2013, 2014) to refer to the participants of this study, although it is not entirely clear that the varieties they are exposed to are Standard Modern Greek and Cypriot Greek or that they are only two varieties, under the assumption that a continuum is in place. For instance, the term ‘Cypriot Standard Greek’ (Arvaniti, 2010) has been proposed to refer to an emerging variety that may count as the standard in the context of Cyprus. This would be a sociolinguistically ‘high’ variety (Ferguson, 1959) that is used in formal settings, although its degree of proximity with Standard Modern Greek is difficult to determine with precision because great fluidity is attested across different settings and geographical areas. At the school environment, for example, one notices the existence of three different varieties: Cypriot Greek, as the home variety that is used when students interact with each other, Standard Modern Greek, as the language of the teaching material, and another standard-like variety that incorporates elements from both varieties, and is present in the repertoire of both the students and the instructors (Sophocleous and Wilks, 2010; Hadjioannou et al., 2011; Leivada et al., 2017).

Observing the existence of different varieties that have boundaries which are unclear as often evidenced between standard and non-standard varieties, the following questions still beg answers: “Is it at all possible to have continuum-external code-switching, if part of Standard Greek is taken to belong to the Cypriot continuum, or if we are dealing with a “fused lect”? How do acquisition factors enter the picture? And, finally, do such data allow us to make a case for competing grammars, and, if so, what is the precise nature of the competition?” (Tsiplakou, 2009).

Importantly, the answers to these questions relate to the study of language variation and posit the question of how the possible existence of functionally equivalent variants fares within a theory of UG that involves parametric values. Functionally equivalent variants in grammar raise the question of constraints, or the lack thereof, on the coexistence of various variants whose distribution is clearly found in different environments when discussed separately. While this is the case for descriptions that focus on the grammar of each variety (Standard and Cypriot Greek) separately, actual use as evidenced through the production of these bilectal speakers reveals a grammar that contains doublets of variants. In certain frameworks, such as the one of Distributed Morphology, the constraints which would explain, for example, the formation of words would focus on the competition of different alternants and the environment in which particular morphemes are inserted in the syntactic component of grammar for spell-out (Embick, 2010, among others). For example, *dur-abil-ity* is a well-formed English word, while ^∗^*dur-ity* is an ill-formed one, suggesting that for this particular case the suffix -*ity* is conditioned by the presence of the morpheme -*able*, which surfaces here as -*abil*. In the current study, grammatical constraints are absent from the variation observed in the variety under investigation. The use of variant A from a doublet (see **Figure 1**) does not prohibit the use of variant B; they cannot appear simultaneously, but they can appear in the same grammatical environment. Our goal here is to document the existence of variation in spontaneous speech and the implications of its existence for variation theories in Universal Grammar.

## Methodology

Typical issues that appear in experimental studies of non-standard varieties relate to the attitude of speakers to present their linguistic repertoire by actively choosing to incorporate characteristics of the standard variety in their speech. This creates problems to experimental investigations that use a variety of structured methodologies, ranging from direct investigation of judgments by speakers, to offline questionnaires and other elicitation techniques. Importantly, spontaneous speech is not necessarily biased for any external factors that relate to speakers’ attitudes and *can* show the different choices available in speakers’ repertoire. In fact, the data presented here that show variation in all the different components of grammar could not be collected in any way other than spontaneous speech: speakers can often choose to use one variant from each doublet, depending on their language attitude toward Cypriot Greek and Standard Modern Greek. Carefully designed experimental studies would probably focus again on the acquisition or use of one part of these pairs and in cases where they consider using both as variables, they can prime and/or guide speakers’ response by either making them aware of the presence of these variables in their speech or, by using specific lexical items that can prime the production of one variant instead of another (Papadopoulou et al., 2014).

For these reasons, the current study presents spontaneous speech data that are important both to the study of this specific variety, but more importantly, to the field of language variation. The next section presents the study by providing the linguistic profile of the participants, and the procedure followed for the analysis variants that we will discuss.

### Participants and Corpus

All participants in this study are neurotypical adults, native speakers of Cypriot Greek. In total, five participants and two researchers interact in five different occasions. The researchers are also adults and native speakers of Cypriot Greek. **Table 1** presents the demographic characteristics of participants’ and researchers’ and the number of utterances produced during each recording. Participants (PA) and researchers (RE) are presented in chronological order with PA1 being the youngest of the participants and PA5 being the oldest. Only female participants were recruited in order to avoid gender effects: previous research that investigated the linguistic production of speakers of Cypriot Greek has identified gender as one relevant factor that affects linguistic performance. More specifically, it has been observed in the relevant literature that male speakers with a particular level of education and degree of familiarity with the researcher show higher rates of use of the Cypriot rather than the standard-like forms (Tsiplakou et al., 2016).

**Table 1:** Participants.

Recording	Participant	Age	Education	Utterances	Total per recording
1	PA1	21	Graduate Degree	333	994
	RE1	25	Post-graduate degree	365	
	RE2	31	Post-graduate degree	296	

2	PA2	21	Graduate Degree	528	999
	RE1	25	Post-graduate degree	208	
	RE2	31	Post-graduate degree	263	

3	PA3	33	Post-graduate degree	385	847
	RE1	25	Post-graduate degree	161	
	RE2	31	Post-graduate degree	301	

4	PA4	54	Secondary Education	315	918
	RE1	25	Post-graduate degree	222	
	RE2	31	Post-graduate degree	381	

5	PA5	57	Secondary Education	647	1060
	RE1	25	Post-graduate degree	177	
	RE2	31	Post-graduate degree	236	
	Mean Age	34,5	Total	4818	4818

*PA4 and PA5 are ‘outliers’ in two respects: they are both older and less educated, with the gap in age and education between them and the other participants being rather significant. Since this is an orientative, small-scale study, we opted for including them in our sample.*

Given that level of education is also found to play a role in the literature —male speakers that have completed secondary education *only* produced forms that were less close to the standard according to Tsiplakou et al. (2016)— we have included in our sample participants with different levels of education.

### Procedure

The purpose of this work is to identify the nature and limits of hybridity (i.e., understood here as the incorporation in one lect of elements that once belonged to different lects probably due to language-dialect contact; a process that results to the existence of functionally equivalent variants in a single lect) in whatever the home variety corresponds to across different speakers. We aim to show that, even in those lects that are closer to the standard, great variation and grammatical hybridity still exists. All participants had a good degree of familiarity with the researchers in order to ensure that the conversation would flow effortlessly. Aiming to obtain a truly spontaneous production, participants had no training in linguistics and no information as to what the researchers were interested in. Participants were familiar with the REs’ profession and they were told that the researchers would like to record a 30-min discussion, without knowing any further details. The recordings took place at participants’ houses and other places that were familiar to the participants.

The seven participants presented in **Table 1** produced 4.818 utterances while engaged in a conversation in an informal setting. Every intelligible unit of speech that was separated by pauses was treated as an utterance (see (1) for an example). There were three participants in each session: One participant and two researchers. Each recording lasted for approximately 30 minutes and there was no specific topic of discussion. Participants were free to lead the discussion and talk about whatever they liked. For this reason, the discussions eventually included different topics across sessions, such as the description of a recent trip to China, the possibilities of applying abroad for a post-graduate degree and aspects of the daily ‘update’ between friends. The overall average utterance production per session was 963,6 utterances (441,6 utterances per participant and 261 utterances per researcher). All conversations were recorded and transcribed by researchers other than RE1 and RE2. Cross-verification of transcription and codification was also done by two other researchers.

### Variants

We analyzed the corpus, focusing on three sets of variants, each of which belongs to a different level of linguistic analysis: (i) syntax is approached through clitic placement which varies in declaratives; it is pre-verbal in Standard Modern Greek and post-verbal in Cypriot Greek, (ii) morphology is examined through the use of the Cypriot Greek diminutive affix -*u* (vs. -*ak* in Standard Modern Greek) and (iii) phonology is examined through the use of the Cypriot-specific post-alveolar affricate /t∫/ which corresponds to the Standard Modern Greek palatal /c/ in the lexical items we examined.

Syntax was approached by identifying an environment where the two varieties differ, namely, clitic placement in declaratives: Cypriot Greek requires enclisis (1), whereas Standard Modern Greek requires proclisis (2) (Terzi, 1999; Agouraki, 2001; Mavrogiorgos, 2013; Neokleous, 2014; among others). While matrix environments are identified as showing enclisis in Cypriot Greek (1), embedded environments headed by certain complementizers can show either proclisis or enclisis (Pavlou, 2016). Some speakers admittedly have a preference toward proclisis or enclisis in embedded clauses and because of this variation, these clauses were also included in the sample.

(1) 𝜃elo to. [Cypriot Greek]
  want.PRES.1SG it.NEU.ACC
  ‘I want it.’
(2) to 𝜃elo. [Standard Modern Greek]
  it.NEU.ACC want.PRES.1SG
  ‘I want it.’

Although certain environments are associated with only one option (i.e., matrix declaratives and enclisis in Cypriot Greek), mixed placement patterns arise to varying degrees in the production of Greek Cypriots even when they converse in the home variety. As Tsiplakou et al. (2016, p. 11) show, one finds in the linguistic repertoire of Greek Cypriots some “pragmatically and conversationally unexpected switch[es]”, where standard-like proclisis surfaces with verbs that bear phonological characteristics of Cypriot Greek. In (3), for example, two instances of enclisis are followed by an instance of proclisis where the clitic attaches to a verb whose phonological form includes the palatoalveolar fricative [∫] which is specific to the Cypriot Greek repertoire and absent from Standard Greek.

(3) ksero to tuto ksero to
  know.1SG it.NEU.ACC this.ACC know.1SG it.NEU.ACC
  to e∫i ma𝜃itis mu.
  it.NEU.ACC have.PRES.3SG student.NOM.SG my.GEN.SG
  ‘I know it, this one, I know it! A student of mine has it.’ (Tsiplakou et al., 2016, p. 11)

At the morphological level, we calculated the occurrences of the Cypriot Greek diminutive suffix -*u* vs the Standard Modern Greek -*ak*. The two suffixes have the exact same meaning and function, but they have slightly different distribution depending on the noun declension; their only difference is with respect to the variety they belong to. The -*u* variant is not an option in Standard Modern Greek. Morphology is of particular interest in the context of our study because it has been argued that structural mixing in the emerging *koiné* (i.e., a variety that incorporates elements from the standard variety but is different from it, like Cypriot Standard Greek) is mostly achieved through morphological choices, while Cypriot phonology and syntax show less hybridity and remain largely intact (Tsiplakou, 2014). Diminution is an extremely productive process of derivation across both varieties (Giannoulopoulou, 2010), hence we take the comparison of the two diminutive variants in our corpus to be a reliable indicator of what has been argued to be the most productive domain when it comes to structural mixing.

Another possible pair of alternants is the future marking that is employed in Cypriot Greek with the periphrastic nonpast tense structure *en na* ([lit. ‘is to’]) and its possible Standard Modern Greek alternant *𝜃a* ‘will’. Even if the two would be found in the same context with a future reading, they could involve a very different structure which is not immediately comparable to each other. Merchant and Pavlou (unpublished) show that the periphrastic structure is different than the future marker ‘will’ used in Standard Modern Greek. For this reason, we do not analyze cases like these that arguably have a very different underlying structure.

Phonology was tested by counting the occurrences of the Cypriot Greek postalveolar affricate variant /t∫/, which would be realized as a palatal /c/ in Standard Modern Greek. For example, the realizations of the conjunction ‘and’ would be *t∫e* in Cypriot Greek and *ce* in Standard Modern Greek. The latter variety lacks the post-alveolar affricate making this one of the most salient differences between the two varieties.

For our analysis, we identified all the indicative clauses that feature a clitic, excluding other syntactic environments where the two varieties do not differ (e.g., imperatives, subjunctives), all the occurrences of the two diminutives regardless of their realization in terms of number and case, and all the realizations of the two phonemes /t∫/ and /c/ in words that are syntactically, semantically, and phonologically the same across the two varieties apart from their difference in the phoneme in question (e.g., as in the case of the conjunction ‘and’).

## Results

**FIGURE 2:**
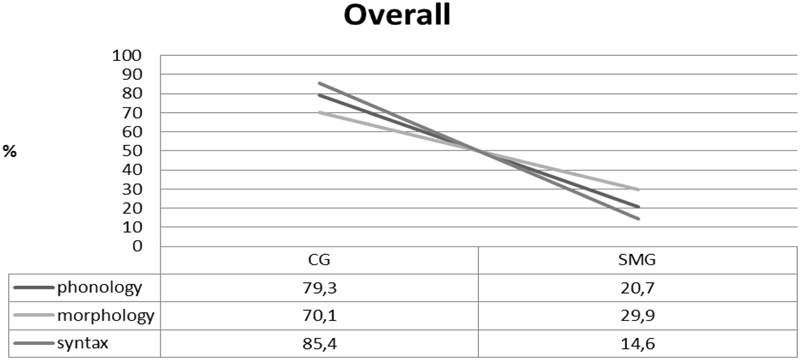
Overall production.

Our findings highlight the presence of variants that belong to different varieties/lects across levels of linguistic analysis. As **Figure 2** shows, the degree of incorporation of elements from one of the different poles of the continuum varies across levels.

**Figure 2** shows that the linguistic repertoire of the participants of this study features predominantly, but not exclusively variants that belong to Cypriot Greek. Morphology indeed stands out as the most hybrid domain (in agreement with what was argued in Tsiplakou, 2014), however some level of hybridity is attested in phonology and syntax as well. This difference was statistically confirmed for all three domains (phonology and syntax X^2^(2) = 19,91, *p* < 0,0001, syntax and morphology X^2^(2) = 10,82, *p* < 0,0001 and morphology and phonology X^2^(2) = 46,75, *p* < 0,0001. In **Table 2**, the number of calculated items is shown for each variety.

**Table 2:** Calculated items.

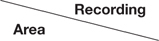	Phonology		Morphology		Syntax		Other utterances (not featuring the variants under examination)
	*t*∫	*c*	*–u*	*–ak*	Enclisis	Proclisis	
**1**	21	15	4	0	39	9	906
**2**	52	5	8	2	79	12	841
**3**	40	3	8	4	99	13	680
**4**	36	7	20	7	88	9	751
**5**	35	18	14	10	58	19	906
**Total**	**184**	**48**	**54**	**23**	**363**	**62**	**4084**

**FIGURE 3:**
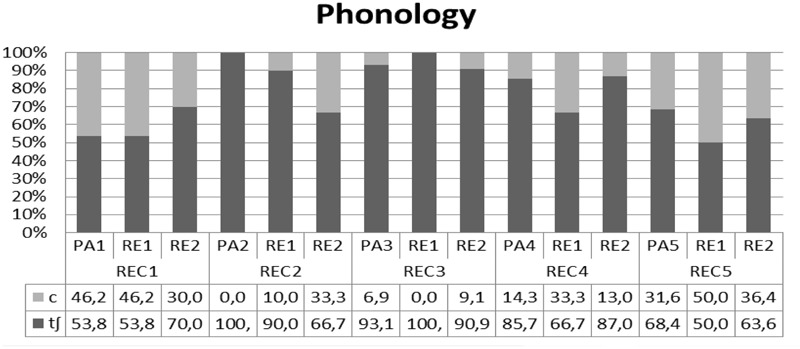
Individual performance for each participant in phonology.

**FIGURE 4:**
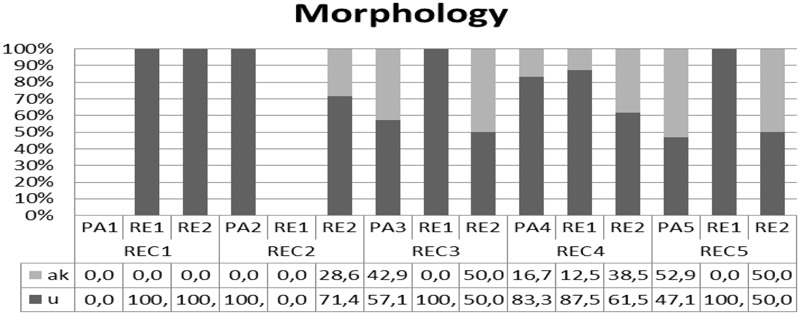
Individual performance for each participant in morphology.

**FIGURE 5:**
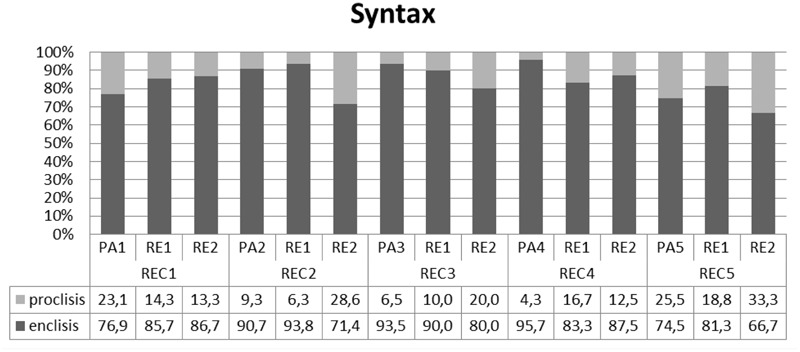
Individual performance for each participant in syntax.

In **Figures 3–5**, the overall performance is broken down for each level of analysis showing the performance of each participant individually. The results reveal the existence of functionally equivalent variants across speakers. In relation to phonology, **Figure 3** shows that all participants, apart from PA2 in recording 2 and RE1 in recording three incorporate both variants to some degree, but prefer the Cypriot /t∫/.

As **Figure 4** indicates, not all participants used diminutives in their spontaneous productions. When diminutives were used, there was a clear preference for the Cypriot Greek variant -*u* rather than the Standard across all participants except RE2 in some sessions.

Syntax is the second domain where all participants incorporated ‘conflicting’ values (i.e., different values of the same variant) of the structures in question in their production. **Figure 5** suggests that the presence of functionally equivalent variants in our bilectal population is not a matter of differential position of each participant on the dialectal continuum. Put differently, our findings reveal both *interspeaker* and *intraspeaker* variation with respect to the patterns of clitic placement that are featured in the grammar under investigation, but with preference for the Cypriot Greek placement pattern.

Participants in our study used different values *without* any code-switching being in place. For example, (4)–(5) were produced by participant PA5 in a succession; consistent enclisis or consistent proclisis could have been used in both cases, but instead she mixed the two throughout her productions and even within the same utterance (5). We thus observe the existence of functionally equivalent variants within her repertoire. Variation is manifested across speakers, as evidenced by the fact that different participants align more with the standard variety than others, but also within speakers, as (4)–(5) suggest.

(4) apla ta ðiakosmisan.
  simply them.NEU.ACC decorate.PAST.3PL
  ‘They simply decorated them.’
(5) ta valan t∫ame ekaman
  ta 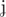
ali.
  them.NEU.ACCput.PAST.3PLthere do.PAST.3PL them.NEU.ACC glass
  ‘They put them there, they cleaned them.’

The observed incorporation of patterns from different lects in one grammar goes far beyond the production of the three variants presented above. In some cases, hybridity extends to the production in a single utterance of two or more Cypriot Greek-specific variants — including variants other than the three pairs that are the focus of this study—, in *varying degrees* across utterances (see **Table 3** and examples (6)–(7)).

**Table 3:** Production of multiple Cypriot Greek-specific variants within the same utterance.

	Variant	Plus 1 item^1^	Plus 2 or more items	Other Cypriot Greek-specific items^2^	None
Phonology	*t∫*	57	9	102	16
	*c*	21	3	18	6

Morphology	*–u*	23	3	24	4
	*–ak*	6	4	10	3

Syntax	enclisis	145	22	166	30
	proclisis	17	7	27	11

*^1^The variants calculated are *t∫* and *c* for phonology, -*u* and -*ak* for morphology, and enclisis and proclisis for syntax.*

*^2^The variants calculated are all other Cypriot Greek-specific variants except the three sets examined so far in this study.*

In (6), we see an utterance produced in Cypriot Greek that involves the Standard Modern Greek diminutives and phonology on the two last nouns *only*. The conjunctive ‘and’ consistently appears both times with the Cypriot Greek /t∫/. On the contrary, its realization varies in (7), which is why we claim the incorporation of elements from different lects varies from production to production. These examples cannot be treated as code-switching for a switch would serve no discourse purpose here. Therefore, we conclude that hybridity in the grammar offers a more accurate description of the situation at hand.

(6) Elpizo na men fao apla t∫e monon 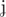
ati exo
  hope.*PRES.1SG* to not eat.*PRES.1SG* simply and only because have.PRES.1SG
  iðieteri a
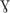
api pros ta 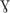
ataca t∫e ta scilaca.
  special love to the kitties and the doggies
  ‘I hope to not eat because I have a special love for kitties and doggies.’
(7) t∫e en:a kamnun opos tin ðania ce ta lipa.
  and FUT do.*PRES.3PL* like the Denmark and the rest
  ‘And they will act like Denmark etc’.

**FIGURE 6:**
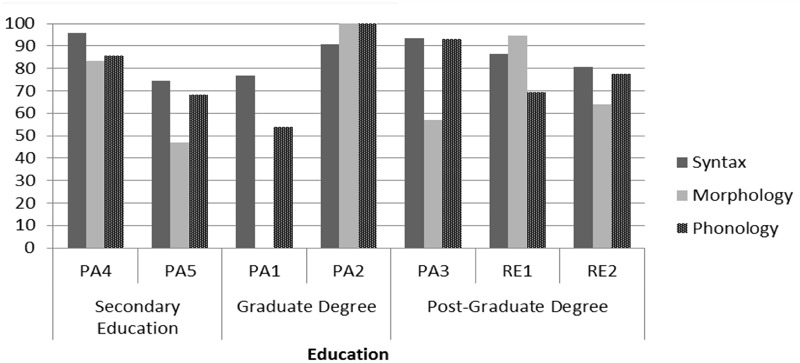
The use of Cypriot Greek variants across participants and levels of education.

**Figure 6** breaks down the performance that features the Cypriot variants of the three pairs under investigation in relation to the different levels of education of the participants. It can be observed that interspeaker variation transcends the boundaries set by different levels of education. For instance, PA4 and PA5 have both completed secondary education only, but their performance is quite different in all levels of analysis.

## Discussion

Comparing the occurrences of three different pairs of variants in a corpus of spontaneous speech, we argue that in the case of our bilectal subjects, the competing grammars that are in place in the process of language acquisition —a claim that is frequently explored in the relevant literature (see Tsiplakou, 2009; Grohmann and Leivada, 2012; Papadopoulou et al., 2014)— eventually result in a linguistic repertoire that involves *mixed* grammars. More specifically, we argue that the existence of (i) closely related varieties that form a dialect-standard continuum and (ii) non-standardization may affect the process of linguistic development and its outcome through blurring the boundaries of linguistic variants. The speaker that once had to deal with ambiguous input and project multiple (competing) grammars has now a repertoire that includes elements from these different grammars/lects into a single grammar. More concretely, the linguistic repertoire of our subjects eventually incorporates values from the acrolect (Standard Modern Greek or Arvaniti’s 2010 ‘Cypriot Standard Greek’) and the basilect (Cypriot Greek), resulting to intermediate lects that, depending on the context, the purpose of the discourse, and the speaker’s attitude toward language, might approximate more ‘Cypriot Standard Greek’.

We aim to put forth two sets of claims with respect to the findings presented above: First, in terms of the notion of mixed or fused grammars and the notion of competition, and second in relation to UG. Starting off from the former, let us restate the set of questions that should be addressed when discussing variation in the grammar under investigation: “Is it at all possible to have continuum-external code-switching, if part of Standard Greek is taken to belong to the Cypriot continuum, or if we are dealing with a “fused lect”? How do acquisition factors enter the picture? And, finally, do such data allow us to make a case for competing grammars, and, if so, what is the precise nature of the competition?” (Tsiplakou, 2009).

In order to address the first question, we employ Auer’s (1999) criteria in order to first distinguish switching from mixing (**Table 4**).

**Table 4:** Basic criteria for distinguishing code-switching from code-mixing (Auer, 1999).

Code-switching	Code-mixing
• Preference for one language at a time	• Does not relate to preferences of speakers
• Possible to describe how code-switching relates to the two codes	• Difficult to find meaning in alternations
• Occurs at major syntactic and prosodic boundaries	• Affects units of any size

Our participants do not show preference for one language at a time, there is no meaning in their alternations, and the grammatical hybridity affects units of any size. For these reasons, we suggest that code-mixing is in place, and not code-switching. The second step is to decide whether the outcome of this mixing amounts to a fused lect or a mixed lect. Auer (1999) suggests that the use of one variety or the other for certain variants and constituents is obligatory in fused lects. Our findings show the exact opposite pattern (see (4)–(5)): The same variant might be realized with two different values in the spontaneous production of our subjects. In this context, we interpret the variation shown in **Figure 2** as language mixing and not as language fusing, since the observed patterns are not stabilized and intraspeaker variation suggests that speakers do have a choice as to which variant they use.

All in all, our results indicate that the linguistic repertoire of our bilectal speakers incorporates elements from different lects across levels of linguistic analysis, resulting to a mixed lect (see also the results of Pappas, 2014 that show *marginal* preferences of Greek Cypriot speakers in terms of proclisis/enclisis following different complementizers). We thus observe a transition from once competing grammars (i.e., competing during the process of language acquisition) to a mixed grammar in the production of adult, neurotypical speakers. Precisely because this mixed grammar is not standardized, it may differ with respect to the degree of mixing that it features from speaker to speaker, from register to register, and from production to production. Eventually, this mixing gives rise to functionally equivalent variants that are the result of bringing into one grammatical system two realizations of the same variant that each comes from a different grammar.

## Implications for Universal Grammar

Showing that a syntactic or a morphological pattern can receive two different values or realizations, under the exact same syntactic conditions, within the production of a single speaker is at conflict with the mainstream conception of our initial state of the faculty of language within a generative approach (i.e., UG). Yang (2004) presents this conflict in the following way: “adult speakers, at the terminal state of language acquisition, *may retain multiple grammars, or more precisely, alternate parameter values*; these facts are fundamentally incompatible with the triggering model of acquisition […] *It is often suggested that the individual variation is incompatible with the Chomskyan generative program*” (2004: 50-51; emphasis added). This alternation between parameter values is evident in the repertoire of our speakers, but also in earlier forms of Greek such as Later Medieval Greek (Pappas, 2004).

As mentioned already, the second aim of the present work is to flesh out the implications that ‘conflicting’ values of functionally equivalent variants carry for parametric approaches to UG. More concretely, in light of the obtained results, our aim is to examine whether there is a way to reconcile the attested variation (as this is manifested both within and across speakers) with UG as one of the main pillars of generative linguistics. We suggest that this is possible. This way entails stripping down UG to only *operations* (see also Di Sciullo et al., 2010 for a claim along these lines). A UG that consists of parameters and parametric values would have trouble explaining how the linguistic repertoire of a neurotypical, adult speaker can involve functionally equivalent variants with different values that are alternatively realized in the *same* syntactic environment. Arguing in favor of microvariation that is sensitive to individual lexical items (as in Kayne, 2005, see also the collection of papers in Eguren et al., 2015) instead of different syntactic environments would not solve the problem at hand, as speakers alternate across values for the exact same lexical item when this is realized multiple times in their production.

A non-parametric theory of UG that encompasses only operations would, however, be compatible with the ‘conflicting’ values of the functionally equivalent variants that are found in the grammar under investigation. Moreover, through showing that the attested patterns of variation in this grammar are not compatible with parametric approaches to UG, we essentially take a step toward removing parameters from the UG inventory. This step would be in the direction of approaching language from below (Chomsky, 2007) through relegating (parametric) variation from UG to the externalization component of language. This idea is increasingly explored in current conceptions of Minimalism (Berwick and Chomsky, 2011; Leivada, 2015; Chomsky et al., unpublished).

The analysis and interpretation of our results shows that there clearly exists a way of investigating some of the contents of UG, hence there exists a way of ‘falsifying’ these contents. Falsification should be understood as subjecting these contents to analysis that confirms or disconfirms our current theory about them. The issue of falsification is important because linguists that question UG have often highlighted in their criticisms the ‘unfalsifiability’ argument (Dabrowska, 2015; Lin, 2017 and references therein). If a theory makes no falsifiable claims, it is an unscientific theory (Popper, 1959), and indeed it would be worrying if a theory of UG involved no falsifiable predictions. We embrace Chomsky’s (1980) view that this is not the case for UG.^4^

Parameters have been traditionally conceived as UG primitives that are part of our innate ability to acquire language; our *language-readiness*, to use Lenneberg’s (1967) term. This theory makes certain predictions about parameters being set to a single value (Chomsky, 1981 et seq.). We have demonstrated the existence of patterns of variation that show different grammatical options (i.e., parametric values) being operative and alternating after the critical period both across and within speakers. This value-alternation possibility suggests that the ‘triggering-a-single-value’ approach is not correct. Such a conclusion inevitably presupposes that our theory about primitives of UG is subject to falsification.

All in all, our results lead us to the claim that points of variation (what is referred to as ‘parameters’ in generative terms) may not be fixed in terms of their values even past the acquisition stage in a neurotypical speaker. Of course, the phonological exponents discussed in the previous section do not bear any relation to parametric variation and cannot support this claim, however, considering the big range of proposals that suggest parametrization of morphosyntax (see Leivada, 2015 for an overview), it is no surprise that clitic placement has been related to parametric variation. One explanation that has been proposed in the literature is that a filled C^0^ requirement gives rise to enclisis (as in Cypriot Greek), while proclisis arises from the absence of this requirement, as happens in Standard Greek (Agouraki, 2001). Clitic placement has thus been explicitly argued to be the outcome of the interplay between the Proclisis Parameter and verb movement (Duarte et al., 2005). This enables us to make the connection between our results and (parametric) theories of UG.

## Conclusion

In collecting and analyzing spontaneous speech data in an understudied variety, we implement two of Hagoort’s (2014) suggestions for maximizing the contribution of linguistics within the greater scheme of things in cognitive science: (i) the use of corpora and (ii) the exploitation of language-specific information which is a “unique selling point of linguistics”. The first aim of this work was to illustrate that grammatical hybridity, understood here as the incorporation of elements from two different linguistic systems into a third linguistic system, results to the existence of functionally equivalent variants across speakers and levels of analysis. We have argued that the once competing grammars that are in place in the process of language acquisition (Grohmann and Leivada, 2012) result in a mixed, hybrid system, that of the adult performance, in which elements from different lects are merged into a single grammar.

The second aim was to show that the patterns of variation attested in this hybrid lect boil down to language mixing, and not fusing or switching. Our results show that indeed mixing takes place, and consequently, the Consensus Principle (Labov, 1996) cannot be straightforwardly assumed as true for speakers of non-standard varieties that acquire language in an environment that involves exposure to a standard-dialect continuum. In view of these findings, we have claimed that only a non-parametric theory of UG is compatible with the ‘conflicting’ values of the functionally equivalent variants that create the grammar under investigation. Last, the noted incompatibility between value-alternation and the ‘triggering-a-single-value’ approach of parametric models, has led to the suggestion that theories of UG are indeed based on falsifiable (or ‘refutable’ to use Chomsky’s, 1980 word) hypotheses, and as such claims about the alleged unfalsifiability of UG should be dismissed as unfounded.

## Ethics Statement

This study has been reviewed by the Cyprus National Bioethics Committee which waived the need for a full screening. The study was conducted in accordance with the declaration of Helsinki and written informed consent was obtained from each participant.

## Author Contributions

EP and NP recruited the subjects and participated in the recording sessions. EP supervised the transcription of the material. EL, EP, and NP analyzed the results. EL drafted the manuscript. EP and NP reviewed and revised the manuscript.

## Conflict of Interest Statement

The authors declare that the research was conducted in the absence of any commercial or financial relationships that could be construed as a potential conflict of interest.

## Footnotes

1 Code-switching refers to the alternation of two languages within a single sentence, constituent, or discourse intersententially and/or intrasententially (the latter is also known as code-mixing; Poplack, 1980; Auer, 1999).

2 In noting the possibility of a discrepancy between judgments and spontaneous production, we do not question the reliability of acceptability judgment tasks as tools of research; a reliability that has been repeatedly shown in the literature, mainly for standard/codified languages (see Sprouse and Almeida, 2012; Sprouse et al., 2013).

3 The concept of “competing grammars” goes back to Kroch (1989, 1994), who proposed that speakers project multiple grammars to deal with ambiguous input. This concept has been explicitly connected to the relation between Standard and Cypriot Greek (Papadopoulou et al., 2014; Tsiplakou, 2014; Grohmann et al., 2017).

4 One finds in the literature concrete suggestions about sets of primitives that form part of UG (see Mendívil-Giró unpublished, for a recent review of related long-standing misinterpretations).
